# Estradiol-driven metabolism in transwomen associates with reduced circulating extracellular vesicle microRNA-224/452

**DOI:** 10.1530/EJE-21-0267

**Published:** 2021-08-03

**Authors:** Barend W Florijn, Jacques M G J Duijs, Maartje Klaver, Eline N Kuipers, Sander Kooijman, Jurrien Prins, Huayu Zhang, Hetty C M Sips, Wendy Stam, Maaike Hanegraaf, Ronald W A L Limpens, Rienk Nieuwland, Bas B van Rijn, Ton J Rabelink, Patrick C N Rensen, Martin den Heijer, Roel Bijkerk, Anton Jan van Zonneveld

**Affiliations:** 1 Department of Internal Medicine (Nephrology), Leiden University Medical Center, Leiden, The Netherlands; 2 Einthoven Laboratory for Vascular and Regenerative Medicine, Leiden University Medical Center, Leiden, The Netherlands; 3 Department of Internal Medicine, Division of Endocrinology, VU University Medical Center, Amsterdam, The Netherlands; 4 Department of Internal Medicine (Endocrinology), Leiden University Medical Center, Leiden, The Netherlands; 5 Department of Cell and Chemical Biology (Section Electron Microscopy), Leiden University Medical Center, Leiden, The Netherlands; 6 Laboratory of Experimental Clinical Chemistry, Department of Clinical Chemistry and Vesicle Observation Center, Amsterdam University Medical Center, Amsterdam, The Netherlands; 7 Department of Obstetrics and Fetal Medicine, Erasmus Medical Center Rotterdam, Rotterdam, The Netherlands

## Abstract

**Objective:**

Sex steroid hormones like estrogens have a key role in the regulation of energy homeostasis and metabolism. In transwomen, gender-affirming hormone therapy like estradiol (in combination with antiandrogenic compounds) could affect metabolism as well. Given that the underlying pathophysiological mechanisms are not fully understood, this study assessed circulating estradiol-driven microRNAs (miRs) in transwomen and their regulation of genes involved in metabolism in mice.

**Methods:**

Following plasma miR-sequencing (seq) in a transwomen discovery (*n* = 20) and validation cohort (*n* = 30), we identified miR-224 and miR-452. Subsequent systemic silencing of these miRs in male C57Bl/6 J mice (*n* = 10) was followed by RNA-seq-based gene expression analysis of brown and white adipose tissue in conjunction with mechanistic studies in cultured adipocytes.

**Results:**

Estradiol in transwomen lowered plasma miR-224 and -452 carried in extracellular vesicles (EVs) while their systemic silencing in mice and cultured adipocytes increased lipogenesis (white adipose) but reduced glucose uptake and mitochondrial respiration (brown adipose). In white and brown adipose tissue, differentially expressed (miR target) genes are associated with lipogenesis (white adipose) and mitochondrial respiration and glucose uptake (brown adipose).

**Conclusion:**

This study identified an estradiol-drive post-transcriptional network that could potentially offer a mechanistic understanding of metabolism following gender-affirming estradiol therapy.

## Introduction

Sex hormones like estrogens have a key role in the regulation of energy homeostasis and metabolism (1). Particularly upon binding the estrogen receptor α (ESR1) or ERβ (ESR2) in adipose tissue, estrogen is known to affect adiposity and insulin sensitivity (2). Also, regular estradiol administration (in combination with antiandrogenic compounds) in transwomen may affect energy metabolism by increasing total body fat (3), fasting insulin, and HOMA of insulin resistance (HOMA-IR) (4), thereby reducing peripheral insulin sensitivity (5). This sex hormone-associated decline in metabolic health increases the risk for type 2 diabetes and future cardiovascular disease (CVD) in transwomen (6). However, the underlying pathophysiological mechanisms are not fully understood.

It has become increasingly clear that post-transcriptional networks through an intricate interplay of non-coding RNAs such as microRNAs (miRs) and RNA binding proteins coordinate the expression of multiple sets of functionally related genes, that together shape the functional response of cells to a change in metabolic demand (7). Also in human adipose tissue, studies have addressed this miR-mediated coordination of gene expression via the complementarity of base pairing at 3′-untranslated regions (3′-UTR) of target mRNAs (8). Interestingly, sex-specific expression of miRs may result via at least two mechanisms: (i) double dosage of X-chromosome located (X-linked) miRs due to incomplete silencing of the X-chromosome and (ii) estrogen regulation of miR transcription and processing (9). Given that increasing evidence is linking estrogen regulation in adipose tissue to whole-body metabolism (10), sex-specific, adipose tissue-derived miRs (11) could potentially alter metabolism at distal tissue sites (12).

Therefore, to understand how trans-hormones (estradiol) associate with post-transcriptional regulation of functionally related genes in metabolism, this study assessed circulating estradiol-driven miRs in transwomen and their regulation of genes involved in metabolism in mice.

## Methods

A full description of the methods can be found in the Supplementary methods (see section on supplementary materials given at the end of this article). Below a summary can be found.

### Patients

Transwomen received oral treatment with a daily dose of both 50 mg cyproterone acetate (CPA) (Androcur^®^, Bayer) and 4 mg estradiol valerate (Progynova®, Bayer) or 100 µg/24 h transdermal estradiol (Systen^®^, Janssen–Cilag) twice a week, as previously described (13). Paired plasma samples were obtained at the start of treatment and after 1 year of daily estradiol administration. Patient characteristics of the transwomen cohort 1 and 2 are displayed in Tables 1 and 2, respectively.

**Table 1:** Clinical characteristics of the pilot *n* = 20 transwomen cohort.

	Baseline	Estradiol	*P*-value
Age (years)	35 ± 13	36 ± 13	
BMI (kg/m^2^)	25.2 ± 4.1	24.5 ± 6.8	0.623
Estradiol (pmol/L)	96 ± 21	310 ± 246	0.001
Testosterone (nmol/L)	19.9 ± 8.7	0.8 ± 0.2	0.001
SBP (mmHg)	133 ± 10	128 ± 12	0.051
DBP (mmHg)	86 ± 11	82 ± 9	0.167
Hemoglobin (mmol/L)	10.2 ± 0.5	9.0 ± 0.2	0.002
Hematocrit (L/L)	0.47 ± 0.02	0.43 ± 0.02	0.004
Glucose (mmol/L)	5.6 ± 0.5	5.5 ± 0.6	0.824
Insulin (pmol/L)	72 ± 44	105 ± 67	0.002
Creatinine (µmol/L)	79 ± 9	79 ± 13	0.913
Cholesterol (mmol/L)	4.59 ± 0.86	4.05 ± 0.71	0.002
Triglyceriden (mmol/L)	1.06 ± 0.42	0.97 ± 0.36	0.303
HDL-cholesterol (mmol/L)	1.38 ± 0.37	1.16 ± 0.30	0.001

HDL, high density lipoprotein; SBP, systolic blood pressure; DBP, diatolic blood pressure.

**Table 2:** Clinical characteristics of the validation *n* = 30 transwomen cohort.

	Baseline	Estradiol	*P*-value
Age (years)	34 ± 12	35 ± 13	
BMI (kg/m^2^)	23.5 ± 6.1	24.9 ± 4.3	0.074
Estradiol (pmol/L)	86 ± 22	275 ± 231	0.001
Testosterone (nmol/L)	20.4 ± 6.3	0.8 ± 0.4	0.001
SBP (mmHg)	127 ± 10	122 ± 9	0.003
DBP (mmHg)	80 ± 9	75 ± 8	0.003
Hemoglobin (mmol/L)	9.8 ± 0.5	8.8 ± 0.5	0.001
Hematocrit (L/L)	0.45 ± 0.03	0.42 ± 0.02	0.005
Glucose (mmol/L)	5.4 ± 0.7	5.2 ± 0.7	0.226
Insulin (pmol/L)	50.1 ± 30.9	71.8 ± 49.4	0.036
Creatinine (µmol/L)	78.2 ± 8.8	73.1 ± 9.0	0.001
Cholesterol (mmol/L)	4.7 ± 1.1	4 ± 0.8	0.001
Triglyceriden (mmol/L)	1.1 ± 0.5	0.9 ± 0.3	0.033
HDL-cholesterol (mmol/L)	1.4 ± 0.3	1.1 ± 0.3	0.001

HDL, high density lipoprotein; SBP, systolic blood pressure; DBP, diatolic blood pressure.

### Plasma RNA isolation

Plasma RNA from each patient sample was isolated from 200 μL EDTA-plasma by using the RNeasy Micro Kit (Qiagen).

### Library preparation and next-generation sequencing of plasma miRs

Plasma miR sequencing was performed by Exiqon according to protocol. Samples were sequenced on the Illumina NextSeq 500 system. Experiments were conducted at Exiqon Services, Denmark.

### Plasma EV isolation

Plasma EVs (70 nm) were isolated by applying 125 μL human plasma to a 3.64 mL Sepharose CL-2B size-exclusion chromatography (SEC) column, as previously described (14).

### qPCR validation of plasma miR, EV miRs, and mRNA expression

Selected miRs were validated with quantitative PCR, using individual samples that comprised the pooled samples that were used for plasma miR sequencing. Taqman primers (Cat. 4427975, Thermo Fisher Scientific) were used according to the manufacturer’s protocols. Target gene mRNA primer sequences are listed in Supplementary Table 2.

### Animal experiments

Male C57Bl/6J mice (*n* = 10 per group, age = 8 weeks, Charles River Nederland) were randomized in three groups and received two s.c. injections of 25 mg/kg locked nucleic acid (LNA)-modified antisense miR-224 (antimiR-224), miR-452 (antimiR-452), or scrambled miR sequence (scramblemiR). Five days before injection, mice were individually housed in fully automated metabolic cages (LabMaster System, TSE Systems).

### Plasma ELISA

Plasma insulin concentrations were measured by ELISA (Mercodia, 10-1247).

### Clearance of radiolabeled glucose and lipoprotein-like particles

Glycerol tri[^3^H]oleate-labeled lipoprotein-like triglyceride (TG)-rich emulsion particles (80 nm) were prepared as previously described (15) and [^14^C]deoxyglucose ([^14^C]DG) was added in a ^3^H:^14^C = 4:1 ratio. After 6 h of fasting, mice were injected with 200 μL of emulsion particles (1 mg TG per mouse) via the tail vein. After 15 min, organs were harvested and dissolved overnight at 56°C in Solvable (Perkin Elmer).

### Tissue histology and immunohistochemistry

Formalin-fixed interscapular BAT (iBAT), s.c.WAT, and gonadal WAT (gWAT) were dehydrated in 70% EtOH, embedded in paraffin, and cut into 5-μm sections. Sections were stained with hematoxylin and eosin (H&E) using standard protocols.

### Mapping and analysis of RNA-seq data

Mus musculus reference version GRCm38.p4 was used for the alignment of samples. The reads were mapped to the reference sequences using a short-read aligner based on the Burrows–Wheeler Transform. The read counts were loaded into the DESeq package v 1.10.1, a statistical package within the R platform v2.15.3. Additionally, RPKM/FPKM (reads/fragments per kilobase of exon per million reads mapped) values were calculated.

### Pathway analysis

Pathway analysis was carried out using Ingenuity Pathway Analysis (IPA) software.

### Cell culture of murine brown adipocytes and 3T3-L1 white adipocytes

Brown preadipocytes were isolated from interscapular BAT depots of 5-week-old male C57BL/6J mice as previously described (16), and upon confluence, cells were differentiated. Experiments were performed on days 12–14 of differentiation. 3T3-L1 preadipocytes (Zenbio) were differentiated in growth medium (DMEM/Ham’s F-12 medium (1:1, v/v) supplemented with HEPES pH 7.4, 10% heat-inactivated FBS (Life Technologies Europe), human insulin, dexamethasone, penicillin and streptomycin, 3-isobutyl-1-methylxanthine (IBMX) and PPARγ agonist rosiglitazone.

### Cell treatment

Differentiated 3T3-L1 adipocytes and differentiated brown adipocytes were incubated with 100 nM 17-β estradiol (E2758, Sigma–Aldrich) for 48 h. After 48 h, cells were washed with PBS combined with Trizol to isolate RNA.

### Oxygen consumption and extracellular acidification of murine brown adipocytes

Oxygen consumption rate (OCR) and extracellular acidification rate (ECAR) were measured using the Seahorse XF96 analyzer (Agilent Technologies).

### Mitotracker experiment

Immortalized murine brown adipocytes were transfected with LNA-antimiR-224 and -452 and incubated for 30 min with MitoTracker Green FM (125 nM; Thermo Fisher) and MitoTracker RedCMXRos (250 nM; Thermo Fisher) in DMEM/F12 (Sigma) without FBS.

### Glucose uptake experiments

Glucose uptake by immortalized murine brown adipocytes transfected with LNA-antimiR-224 and -452 was performed using a glucose uptake colorimetric assay kit (Sigma–Aldrich/Merck) according to the manufacturer’s protocol.

### Statistical analysis

Differential expression analysis of plasma miRs in the next generation sequencing experiment was done using the EdgeR statistical software package (Bioconductor). For normalization, the trimmed mean of the M-values method was used based on log-fold and absolute gene-wise changes in expression levels between samples (TMM normalization). All other data are expressed as mean ± s.e.m. Variable distribution was tested using the Kolmogorov–Smirnov test for normal distribution. In addition, multivariable linear regression was used to adjust for possible confounders. Statistical analysis was performed with GraphPad software using a two-tailed paired or unpaired Student’s *t*-test or ANOVA with Bonferroni’s* post hoc* test.

### Study approval

These studies were approved by the Institutional Review Boards of both the VU University Medical Center (Amsterdam, The Netherlands) and the Leiden University Medical Center (Leiden, The Netherlands) and complied with the ethical principles of the Declaration of Helsinki. All patients gave written informed consent. All animal experiments and protocols were approved by the animal welfare committee of the veterinary authorities of the Leiden University Medical Center (Leiden, The Netherlands).

### Data and resource availability

The datasets generated during and/or analyzed during the current study are available in the Gene Expression Omnibus (GEO) and are accessible under GSE147966 (reviewer token for access: ahmfqeqglzwphcf). No applicable resources were generated or analyzed during the current study.

## Results

### Identification of circulating estradiol-responsive miRs in transwomen

**Figure 1:**
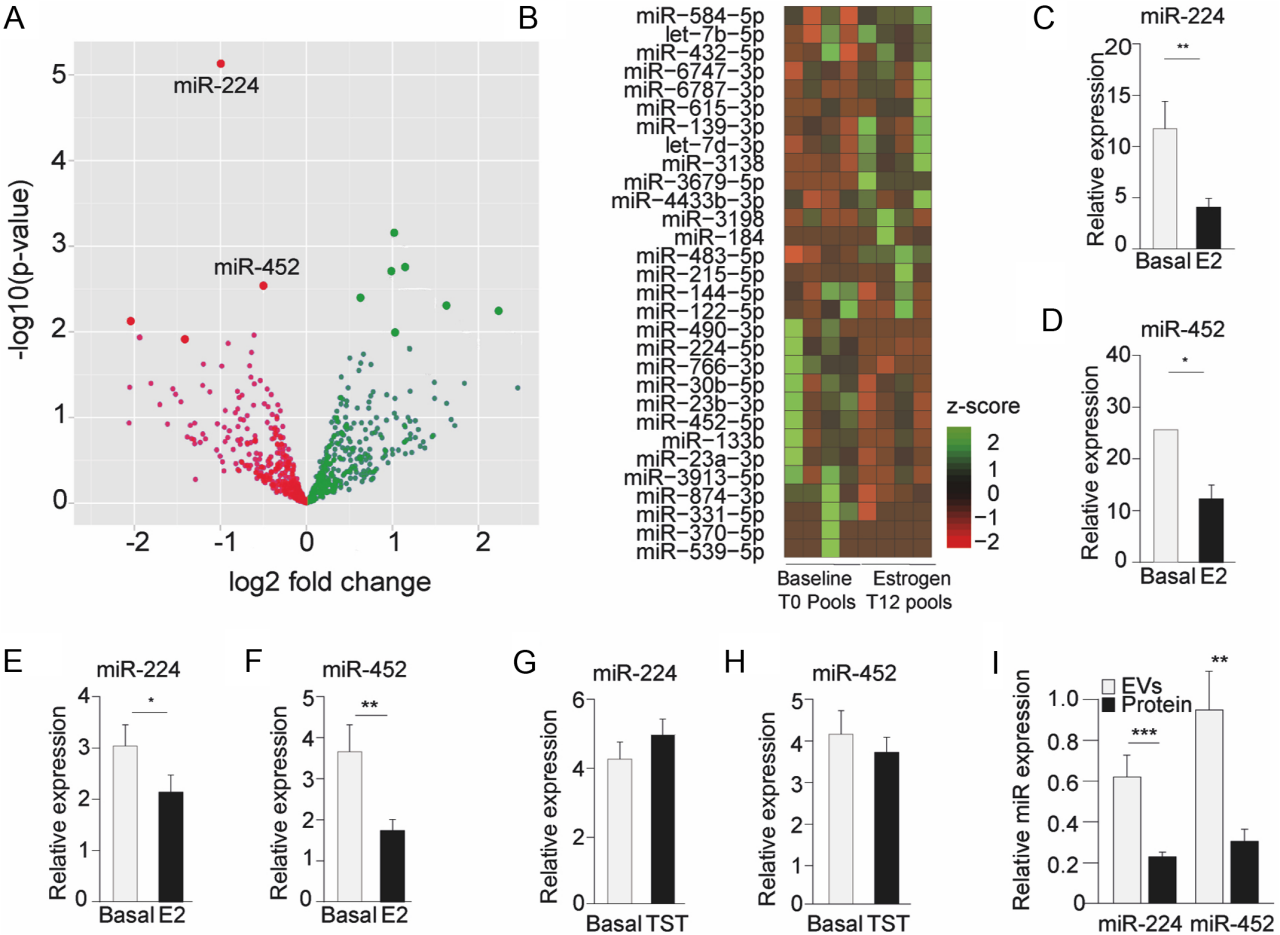
Identification of circulating miRs in human plasma of male-to-female transgender persons (transwomen). (A) Volcano plot depicting differentially expressed miRs after estrogen treatment. (B) Heatmap depicting differentially expressed estrogen-responsive miRs. (C and D) RT-qPCR validation of the differentially expressed miR-224 and miR-452 in the same cohort of 20 transwomen. (E and F) RT-qPCR validation of miR-224 and miR-452 in a second, independent transwomen cohort (*n* = 39). (G and H) Expression levels of circulating miR-224 and miR-452 in transmen after testosterone treatment (*n* = 51). (I) Higher expression levels of miR-224 and miR-452 in extracellular vesicles (EVs) compared to total plasma protein both at baseline and after estrogen suppletion in transwomen (*n* = 20). Basal, baseline state before hormone suppletion; E2, estradiol; TST, testosterone. Data are represented as means ± s.e.m. *P ≤ 0.05, **P ≤ 0.01, ***P ≤ 0.001 according to the two-tailed paired and unpaired Student’s *t*-test.

We performed a pilot study assessing plasma miR profiles in transwomen who received 1 year of estradiol suppletion prior to surgical transition (17). Next-generation sequencing of plasma miRs in four pooled EDTA-plasma samples (pooled based on age and estradiol concentration) from five transwomen, before (*n* = 20) and after (*n* = 20) 1 year of estradiol treatment, identified 667 miRs (Fig. 1A) of which 33 were differentially expressed (Supplementary Data 1). Specifically, miR-224, -122-5p, -539-5p, 23a-3p, -133b, -452-5p, 23b-3p, -3913-5p, -144-5p, -331-5p, -766-3p, -874-3p, 30b-5p, and miR-490-3p levels displayed a significant decrease, while circulating levels of miR-3138, -215-5p, -483-5p, -let-7b-5p, -6787-3p, -184, -3679-5p, -370-5p, -615-3p, -let-7d-3p, -432-5p, -139-3p, -6747-3p, -433b-3p, -584-5p, -3198, -3940-3p, -625-3p, and miR-6786-3p increased (Fig. 1B). The following miRs were selected for RT-qPCR validation: miR-224, -122, -23a, -452, -139, -133b, -215, -9, -874, -30b, -483, -539, and -652. These miRs were selected based on (i) a sufficient level of regulation across sample groups (>2-fold up- or down-regulation, corresponding to +/− 1.0 log-FoldChange) and (ii) differential expression (based on *P*-value) as a result of 1-year estradiol treatment.

### Validation of estrogen-responsive miRs in independent transgender cohorts

To validate these circulating miRs, we conducted a three-step validation process. First, we reassessed plasma miRs (Supplementary Fig. 1) by RT-qPCR in the individual samples of the same transwomen cohort that was used in the pilot study, before (*n* = 20) and after (*n* = 20) estradiol suppletion (Table 1 for clinical characteristics). Out of the 13 selected miRs, we confirmed a significant decrease in circulating levels of miR-224 (Fig. 1C), miR-452 (Fig. 1D), and miR-133b (Supplementary Fig. 1). Subsequently, in a second independent transwomen cohort (Patient characteristics in Table 2), we again assessed plasma levels of miR-224, miR-452, and miR-133b and demonstrated decreased plasma miR-224 and miR-452 (Fig. 1E and F) while plasma miR-133b was not affected (Supplementary Fig. 2). Because next to estradiol treatment, transwomen also received a daily dose of cyproterone acetate (CPA) to block testosterone secretion, we sought to exclude testosterone effects on the observed differences in plasma miRs. Therefore, we assessed plasma miR-224, -452, and -133b in female-to-male transgender persons (transmen, *n* = 50, clinical characteristics in Supplementary Table 1) after 1 year of testosterone treatment. In transmen, these miRs were not affected (Supplementary Fig. 3). The supposedly coordinated regulation of miR-224 and miR-452 by estradiol could be consistent with the fact that both miRs are transcribed from a single transcript from the GABRE locus on the X-chromosome (Source: miRbase version 21). Following the above and given that both miRs are predominantly expressed in adipose tissue (18), we subsequently focused our experiments on both miR-224 and miR-452. We next assessed whether miR-224 and miR-452 associate with EVs or plasma proteins and used size-exclusion chromatography (SEC, Supplementary Fig. 4) to isolate EVs from plasma of the transwomen pilot study cohort (*n* = 20). This identified an approximately three-fold higher expression of both miRs in EVs compared to the plasma protein fraction (Fig. 1I). Lastly, we examined the correlation of these miRs with the calculated homeostatic model assessment for insulin resistance (HOMA-IR) in transwomen to assess their association with metabolism. Particularly miR-224 displayed a negative correlation with HOMA-IR (*R* = −0.38, *P*  = 0.03, while miR-452 and HOMA-IR were not significantly correlated (data not shown).

### Systemic silencing of miR-224 and miR-452 in mice affects adipocyte-specific nutrient uptake

**Figure 2:**
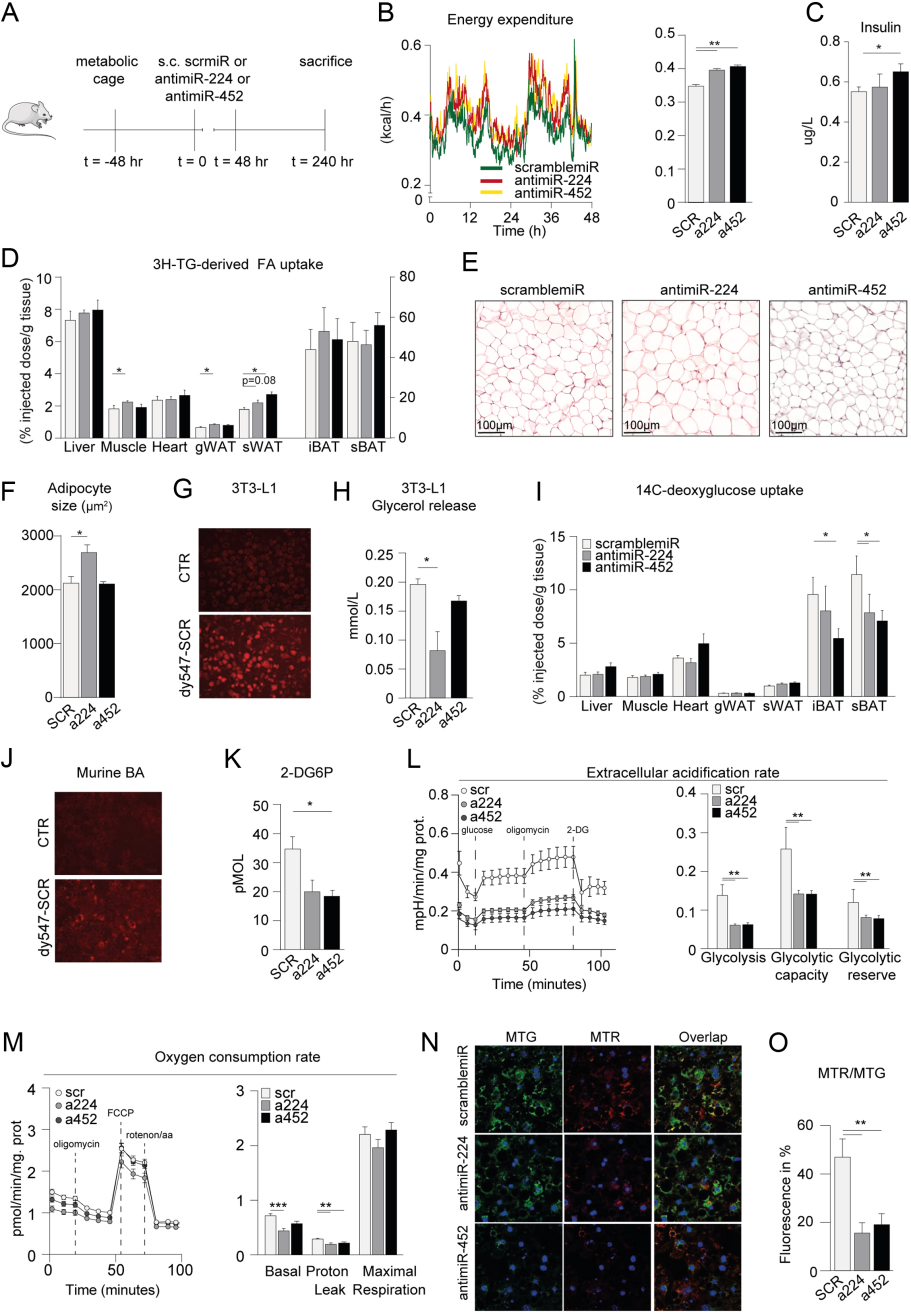
Systemic silencing of miR-224 and miR-452 affects adipocyte-specific nutrient uptake. (A) Experimental set up, mice were housed in calorimetric cages 48 h before i.p. injection of scramblemiR (*n* = 10), antimiR-224 (*n* = 10), and antimiR-452 (*n* = 10). 240 h after injection mice were sacrificed. (B) Energy expenditure (EE) was assessed and quantified over 48 h. (C) miR-452 silencing increased plasma insulin levels. (D) miR-224 silencing increased triglyceride-derived fatty acid uptake in skeletal muscle, gonadal WAT (gWAT), and s.c.WAT, while miR-452 silencing increased triglyceride-derived fatty acid uptake in s.c.WAT. (E and F) Hematoxylin and eosin (H&E) staining of white adipocyte size in antimiR-224 and antimiR-452 treated mice compared to scramble control mice. (G) Transfer of dy547-labeled siRNAs into male 3T3-L1 white adipocytes. (H) Decreased glycerol release in culture media of antimiR-224 treated 3T3-L1 white adipocytes (*n* = 6) only. (I) miR-224 silencing reduced deoxyglucose uptake in subscapular BAT (sBAT), while miR-452 silencing decreased deoxyglucose uptake in interscapular BAT (iBAT) and subscapular (sBAT). (J) Transfer of dy547-labeled siRNAs into murine male immortalized brown adipocytes. (K) Decreased glucose uptake in antimiR-452 treated immortalized brown adipocytes (*n* = 4–6). (L) Decreased extracellular acidification rate (ECAR) of antimiR-224 and antimiR-452 treated murine male immortalized brown adipocytes (*n* = 10) followed by its quantification. (M) Oxygen consumption rate (OCR) of antimiR-224 and antimiR-452 treated murine male immortalized brown adipocytes (*n* = 10) followed by its quantification. (N) Representative images of antimiR-224 and antimiR-452 treated, immortalized brown adipocytes (*n* = 4–6) stained with MitoTracker Green FM (125 nM) and MitoTracker Red CMXRos (250 nM). Fluorescence of MitoTracker stained cells was imaged using a confocal laser scanning microscope (Leica TCS SP8, Leica Microsystems). (O) Quantification of MitoTracker Green (MTG) and MitoTrackers Red (MTR) using ImageJ. SCR, scramblemiR; a224, antimiR-224; a452, antimiR-452. Data are represented as means ± s.e.m. *P ≤ 0.05, **P ≤ 0.01 according to a one-way ANOVA, Bonferroni’s* post-hoc* test.

To investigate the function of miR-224 and miR-452, we set up a mouse study allowing silencing these miRs to study its impact on lipid and glucose metabolism. First, male C57BL/6J mice (*n* = 10) were housed in metabolic cages and injected with locked nucleic acid (LNA) antisense (anti)-miR-224, LNA antimiR-452, or control scramblemiR (Fig. 2A). Within 48 h, antimiR-224 and antimiR-452 injection in mice elevated energy expenditure (Fig. 2B) although body weight, respiratory exchange ratio, carbohydrate oxidation, and fat oxidation were not significantly altered (data not shown). Given the altered energy expenditure we studied whether plasma insulin levels were affected and found that in antimiR-452 treated mice, insulin levels were significantly increased after 48 h (Fig. 2C). Therefore, to study the effect of both miRs on nutrient uptake, we next assessed triglyceride (TG)-derived fatty acid (FA) uptake in metabolic tissues such as skeletal muscle and adipose tissue, using glycerol tri[^3^H]oleate packaged in TG-rich lipoprotein-like particles. In antimiR-224 treated mice, [^3^H]oleate uptake was significantly increased in skeletal muscle and gonadal white adipose tissue (gWAT), and a trend toward increased [^3^H]oleate uptake was observed in s.c.WAT(*P*  = 0.08), while antimiR-452 treatment of mice increased [^3^H]oleate uptake in s.c. white adipose tissue (Fig. 2D). To study whether the observed increase in [^3^H]oleate uptake led to an increase in white adipocyte size, we performed HE staining of s.c.WAT tissue, which demonstrated a marked increase in white adipocyte size in the antimiR-224-treated mice (Fig. 2E and F). *In vitro*, in male murine 3T3-L1 adipocytes, miR-224 silencing (Fig. 2G indicates fluorescent signal detection upon dy547-labeled siRNA transfection indicating successful LNA transfer in these cells) similarly affected lipid uptake given the decrease in glycerol release into the culture medium (Fig. 2H) thereby confirming our findings* in vivo*. Subsequently, we studied *in vivo* whether miR silencing altered tissue-specific glucose uptake in mice by injecting [^14^C]deoxyglucose (DG). AntimiR-224 treatment reduced [^14^C]DG uptake in subscapular brown adipose tissue (sBAT), while antimiR-452 treatment reduced [^14^C]DG in both interscapular brown adipose tissue (iBAT) and sBAT (Fig. 2I). Next, in cultured brown adipocytes, we found less glucose uptake following miR-452 silencing only (Fig. 2K) (following confirmation of successful LNA transfer by fluorescent signal detection in brown adipocytes in Fig. 2J). In addition, we observed less glycolysis, glycolytic capacity, and glycolytic reserve in antimiR-224/452 treated cells as determined with Seahorse respirometry (Fig. 2L). Because glucose consumption and glycolytic flux maintain mitochondrial respiration (19), we also measured mitochondrial respiration by proton flux in the culture media of antimiR-224 and antimiR-452 treated brown adipocytes. Although maximal respiration was not significantly affected, we observed a decrease in basal respiration in antimiR-224 cells and a decrease in proton leak in antimiR-224 and -452 treated cells (Fig. 2M). Next, we stained the mitochondria in antimiR-224 and -452 treated murine brown adipocyte cell cultures using Mitotracker green (MTG; total) and Mitotracker red (MTR; active) (Fig. 2N), which stain mitochondria independent of- and dependent on membrane potential, respectively. After quantification of relative fluorescence intensity (MTR/MTG), which measures mitochondrial depolarization, a decrease in mitochondrial depolarization was seen in antimiR-224 and -452 treated cells (Fig. 2O).

### miR-224 and miR-452 target genes are involved in mitochondrial energy metabolism, insulin signaling, glucose metabolism, and lipogenesis

**Figure 3:**
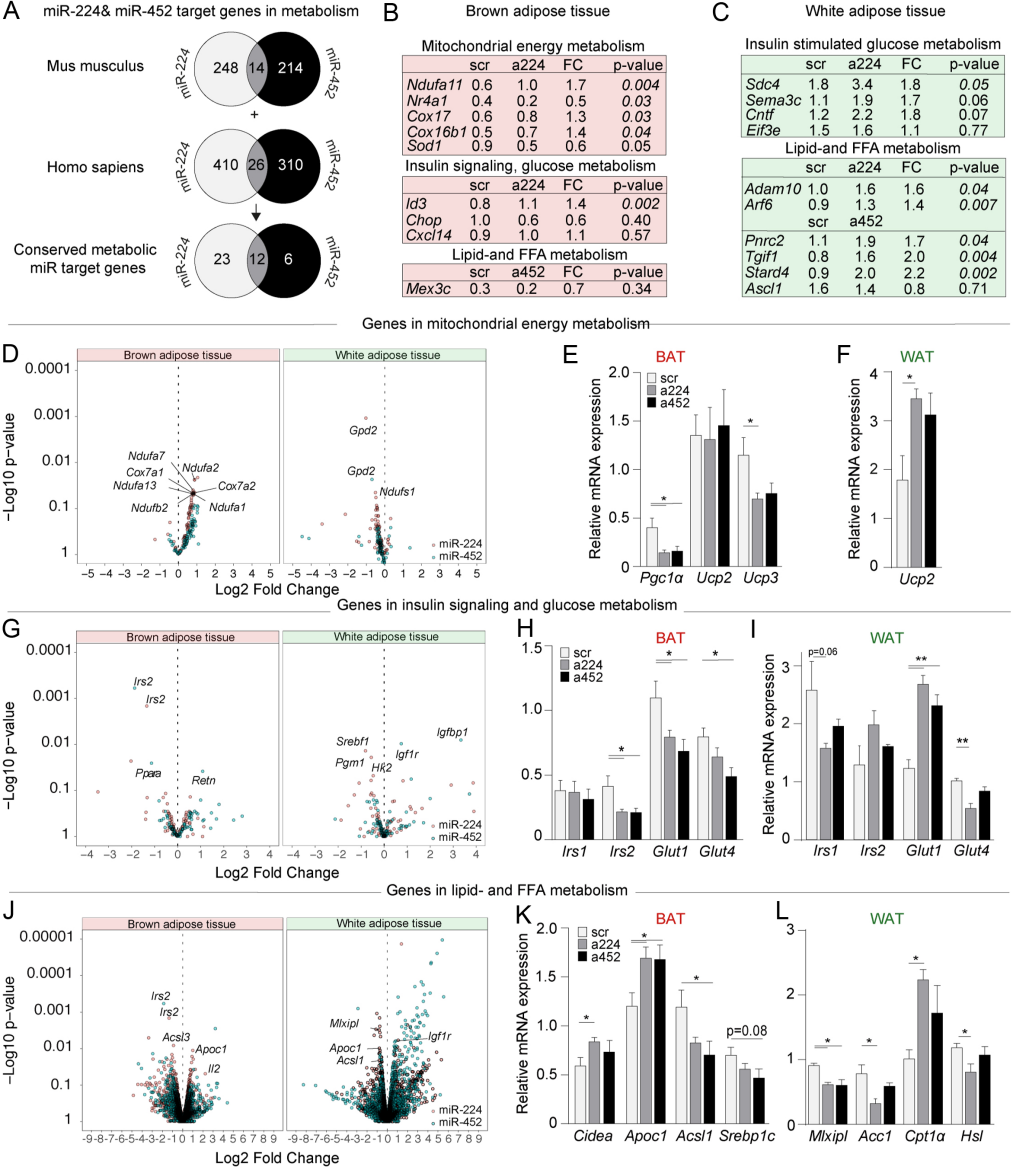
Next-generation sequencing (NGS)-derived differential expression and qPCR validation of (miR target) genes involved in mitochondrial energy, glucose, and lipid metabolism. (A) Both miR-224 and miR-452 have a strong bias toward target genes that associate with metabolism in general. (B) Differentially expressed miR-224- and miR-452 target genes involved in mitochondrial energy metabolism, insulin signaling, glucose metabolism, and lipid metabolism in BAT and (C) WAT tissue. (D) Volcano plot of NGS-derived differential expression of genes (*n* = 4 per group) involved in mitochondrial energy metabolism. (E) RT-qPCR validation (*n* = 8–10 per group) of NGS derived, differential expression of genes involved in mitochondrial energy metabolism in BAT and (F) WAT. (G) Volcano plot of NGS-derived differential expression of genes involved in insulin signaling and glucose metabolism followed by RT-qPCR validation of several key genes in (H) BAT and (I) WAT. (J) Volcano plot of NGS-derived differential expression of genes involved in lipid metabolism followed by RT-qPCR validation in (K) BAT and (L) WAT. Data are represented as means ± s.e.m. *P ≤ 0.05, **P ≤ 0.01 according to a one-way ANOVA, Bonferroni’s* post-hoc* test.

Given that silencing of these miRs stimulated fatty acid uptake in WAT and decreased glucose uptake in BAT, we next performed RNA sequencing (RNA-seq) of WAT and BAT to identify metabolism-associated gene expression changes. Interestingly, using Ingenuity Pathway Analysis (IPA), we found that both miR-224/452 target genes are strongly enriched for genes that associate with metabolism, potentially indicating a coordinated regulation of multiple metabolic pathways by these miRs (Fig. 3A). By further applying *in silico* analysis of the RNA-seq data with the IPA tool, we identified mitochondrial dysfunction and insulin receptor signaling in BAT as significant top canonical pathways affected (Supplementary Fig. 5). In contrast, in WAT tissue from antimir-224 treated mice, AMP-activated protein kinase (AMPK) signaling (regulates adipocyte metabolism) and signal transducer and activator of transcription 3 (STAT3) (controls lipogenesis and adipocyte hypertrophy) were affected, while antimiR-452 treatment affected the HIPPO-pathway (adipocyte differentiation) and insulin receptor signaling. We subsequently used IPA to identify all potential miR-224/452 target genes that could serve as upstream regulators of differential top canonical pathways. When miR-224/452 target genes were plotted in heatmaps, many of them were upregulated in BAT and WAT (Supplementary Fig. 6, metabolic genes marked with an asterisk), and some key metabolism-related target genes could be validated by qPCR (Fig. 3B and C). Specifically, we identified an increased expression of the miR-224 targets *Ndufa11*, *Cox17*, *Cox16b1,* and *Id3* in BAT (Fig. 3B), which associate with metabolic syndrome and obesity (20, 21). *Nr4a1* expression, an effector of BAT thermogenesis (22), was decreased with miR-224 silencing. Interestingly, in WAT (Fig. 3C), antimiR-224 treatment coordinately upregulated genes that regulate lipid metabolisms such as *Arf6* (23, 24) and Adam10 (25) or exacerbate insulin sensitivity like *Sema3C* (26) and *Sdc4* (27). Similarly, antimiR-452 treated WAT tissue displayed higher expression of lipogenesis-associated genes, such as *Pnrc2* (28) and *Stard4* (29) as well as genes that promote insulin resistance like *Cntf* (30) or associate with weight gain and adiposity (*Tgif-1*) (31, 32).

### Adipose tissue-specific differential expression of genes involved in metabolism following miR-224 and miR-452 silencing

Next, we aimed to identify differential expression of genes that function in the top altered canonical metabolic pathways. To that end, we first generated gene-distribution volcano plots confirming differential expression of genes in mitochondrial energy metabolism (Fig. 3D), insulin signaling and glucose metabolism (Fig. 3G), and lipid metabolism (Fig. 3J) (RNA-seq data can be found in Supplementary Data 2, while a selected subset of differentially expressed genes involved in these pathways is displayed in Table 3). Then we validated the differential expression of several genes whose functions are known to have a major impact on energy metabolism by RT-qPCR. With regard to mitochondrial energy metabolism in BAT, qPCR validation confirmed a reduced expression of *Pgc1α* (Fig. 3E and F), which regulates mitochondrial biogenesis and whose loss predisposes to insulin resistance (33). Given the *in vivo* decrease in BAT-specific glucose uptake, we also validated the loss of BAT-specific glucose uptake genes namely *Glut4* and *Irs2* (34, 35) (Fig. 3H). Similar decreases in key insulin signaling genes like *Irs1* and *Glut4* were observed in antimiR-224 treated WAT (Fig. 3I). Finally, in lipid metabolism in BAT, we validated a lower expression of *Cidea* (Fig. 3K), which associates with lipogenesis (36), a decreased expression of *Acsl1*, a promoter of FA oxidation (37), and increased *Apoc1* expression. In WAT-specific lipid metabolism, the expression of *Mlxipl*, *Acc1,* and *Hsl* was decreased (Fig. 3L), which collective decrease is associated with more FA uptake (38, 39, 40).

**Table 3:** Next-generation sequencing derived differential gene expression (FPKM, *P*  < 0.05, *n* = 4) of a selected set of key-metabolism genes involved in glucose and lipid metabolism in brown and white adipose tissue after systemic silencing of miR-224 and miR-452 in mice.

	Glucose metabolism	Lipid metabolism	Fatty acid metabolism	Mitochondrial energy metabolism
BAT				
AntimiR-224 - BAT	*Irs1*↓, *Irs2*↓, *Eif4ebp1*↑, *Frs2*↓, *Jun*↓, *Fbp1*↑, *Pik3ca*↑	*Cidea*↑, *Fabp4*↑, *Acaa2*↑, *Apoc1*↑, *Pdhb*↑, *Pdk4*↓, *Idh3b*↑, *Suclg1*↑, *Acaa1a*↑, *Acca2a*↑, *Acat1*↑, *Acadvl*↑, *Acsl3*↓	*Fabp5*↑, *Slc27a3*↓	*Ppargc1a*↓, *Cox5b*↑, *Cox6b1*↑, *Cox6c*↑, *Cox7a2*↑, *Cox7a2*l↑, *Cox7b*↑, *Cyc1*↑, *Ndufa1*↑, *Ndufa11*↑, *Ndufa2*↑, *Ndufa3*↑, Ndufa4↑, Ndufa6↑, *Ndufa7*↑, *Ndufa8*↑, *Ppa2*↑, *Sdhb*↑, *Slc25a21*↓, *Ucp3*↓,
AntimiR-452 - BAT	*Insr*↓, *Irs2*↓, *Pfkfb3*↓, *Sorbs1*↓, *Pdpk1*↓, *Pir3cb*↓, *Prkc1*↓, *Srebf1*↓, *Sorbs1*↓, *Frs2*↓	*Scd2*↓, *Srebp1c*↓, *Acsl1*↓	*Prkaa1*↓, *Prkacb*↓	*Ppargc1a*↓, *Atp6voa2*↓, *Cox6c*↑, *Lhpp*↑, *Slc25a15*↓, *Ucp2*↑
WAT				
AntimiR-224 - WAT	*Igf1r*↑, *Grb2* ↑, *Ptpn1*↑, *Ptprf*↑, *Adra1d*↓, *Srebf1*↓, *Glut4*↓, *PI3K*↑, *mTOR*↓	*Gpr120*↓, *Mlxipl*↓, *Acc1*↓, *Acc2*↓, *Srebp1c*↓, *Hsl*↓, *Acsl1*↓, *Apoc1*↓	*Acads*↓, *Acsl5*↑, *Cpt1a*↑, *Crat*↓, *Hadha*↓, *Lipe*↓, *Hmgcs2*↑	*Ucp2*↑, *Cox7a2l*↑, *Gadd45b*↑
AntimiR-452 - WAT	*Gys1*↓, *Fbp2*↑, *Igf1r*↑, *Igfbp1*↑, *Ptprf*↑, *Araf*↑	*Gpr120*↓, *Mlxipl*↓, *Acc1*↓, *Apoc1*↓	*Slc27a2*↑, *Prkacb*↑	*Slc25a15*↑, *Slc25a21*↑

## Discussion

**Figure 4:**
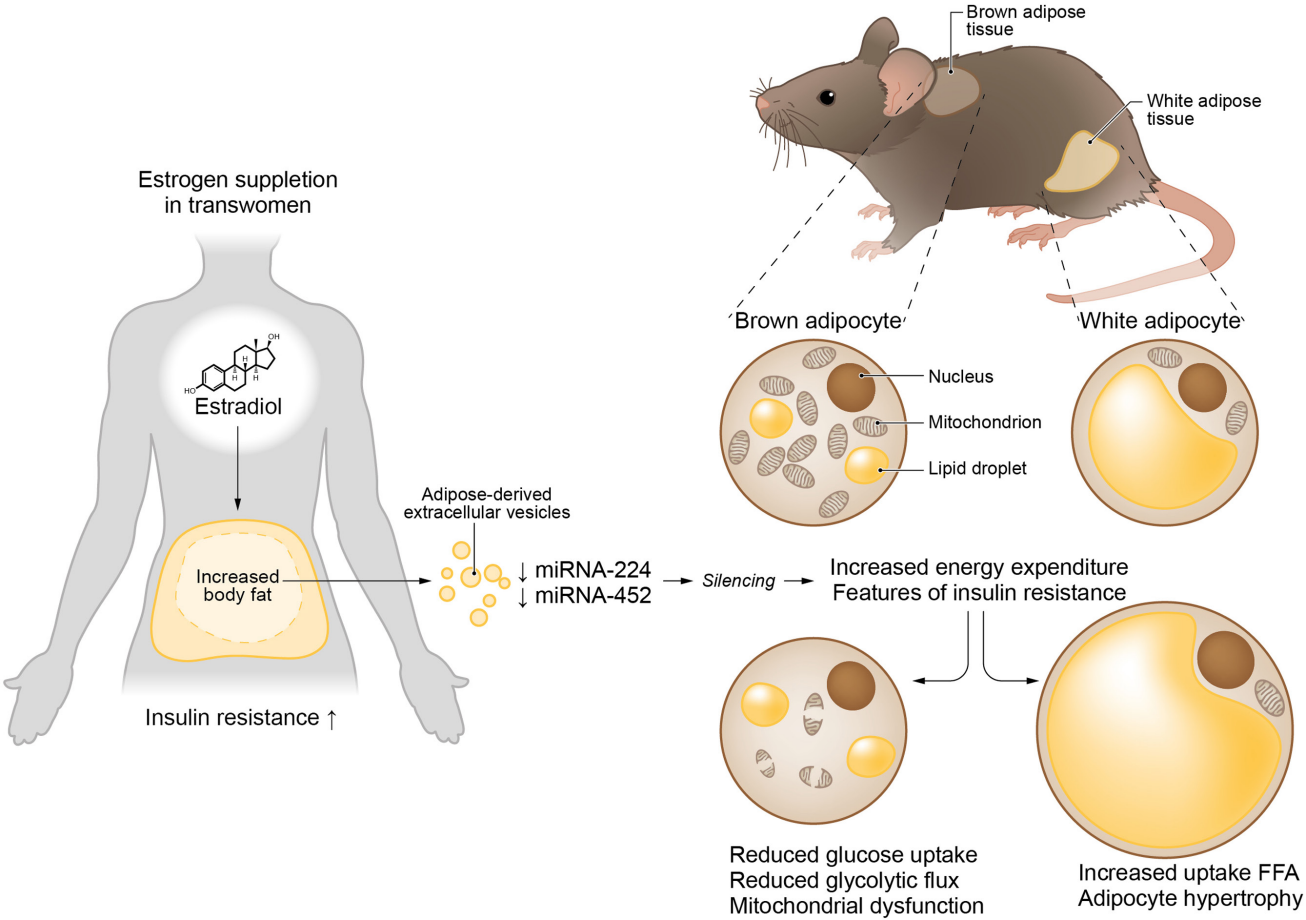
Proposed mechanism by which miR-224 and miR-452 affect metabolism in transwomen. Tightly balanced glucose uptake in brown adipose and lipid uptake in white adipose in lean metabolism is disrupted upon supraphysiological levels of estrogen that lower miR-224 and miR-452 in extracellular vesicles. The loss of both miRs lowers mitochondrial respiration and glucose uptake in brown adipose and results in more triglyceride-derived fatty acid uptake in white adipose and skeletal muscle.

This study demonstrates that estradiol in transwomen lowers circulating miR-224 and -452 carried in extracellular vesicles. Following systemic silencing of these miRs in mice and cultured adipocytes, lipid uptake was increased in skeletal muscle and WAT, while glucose uptake and mitochondrial respiration were decreased in BAT. Differentially expressed genes in these tissues were involved in mitochondrial energy metabolism, glucose uptake, and lipogenesis. As such, this study identified novel estradiol-driven post-transcriptional networks that could potentially offer a novel mechanistic understanding of metabolism following gender-affirming therapy (Figure 4

).

In transwomen, it remains challenging to separate estradiol administration effects from androgen withdrawal effects. Nonetheless, this estradiol-mediated repression of the miR-224/452 cluster was demonstrated before in ER-positive breast cancer patients (41) and is consistent with the idea that miR clusters tend to be regulated in a similar way (42). Moreover, the female genetic background makes transmen not the most suitable control group to exclude testosterone withdrawal effects on these miRs. Still, in women, we previously demonstrated increased circulating levels of miR-224/452 in contrast to no significant effects in the transmen cohort (Fig. 1G and H) (43).

It could be further argued that transmen do not distinguish causal effects of estradiol from CPA treatment nor the fact that a change in androgen/estrogen ratio could alter miR levels. Therefore, different patient cohorts like men receiving androgen withdrawal as prostate cancer treatment or hypogonadal men before and after initiation of testosterone replacement therapy should serve as control groups. However, such particular patients have other confounding characteristics that could affect these miRs. For instance, the Gabre-miR-224/452 locus is downregulated and hypermethylated in prostate cancer patients (44) while low testosterone levels following hypogonadism almost always correlate with the presence of diabetes mellitus (DM) (45). It is also important to note that CPA could have slight glucocorticoid effects as well (46). As such it could potentially affect glucose homeostasis as well which could impact the results of this study. Instead of studying transwomen treated with a combination of CPA and estradiol, more studies are needed to assess the impact of miRs on metabolism. For instance, in transwomen who receive other gonadotropin-releasing hormone receptor (GnRHR) agonists like leuprolide acetate in combination with estradiol, which was found to have no effect on insulin and glucose levels compared to CPA and estradiol (47). However, these sex hormone-mediated effects on the metabolism have been described for estradiol as well (47) while our studies in mice demonstrate significant miR-mediated effects on glucose uptake and mitochondrial energy metabolism. Interestingly, when we performed IPA analyses of the differential proteome of omental adipose tissue from women with gestational diabetes mellitus (GDM) compared to adipose tissue from control subjects (48) we found that mitochondrial dysfunction topped the list of differentially expressed pathways (data not shown) further supporting the relationship between high levels of estradiol and metabolic changes. Lastly, body composition may affect metabolic health and as such may alter miR plasma levels as well (49), although BMI was not significantly affected in transwomen in this study (Tables 1 and 2). Still, future studies should investigate miR-224/452 in relation to body composition changes over time.

We conducted miR-224 and -452 silencing in male mice, particularly because we identified both miRs in male-female transgenders. It could be argued that testosterone signaling in these mice may have confounded *the in vivo* data, while female mice, in which estrogen signaling is intact, would be more appropriate for direct testing of our hypothesis. However, it has been demonstrated that particularly female rats were previously found to become obese and showed impaired systemic insulin sensitivity following mitochondrial dysfunction in BAT, suggesting the potential of sex-specific miR effects in BAT tissue (50). Our observations in mice regarding increased energy expenditure and increased fatty acid uptake could also indicate beneficial effects to prevent lipotoxicity. Although we do not have a direct explanation for the elevated energy expenditure, we suspect that the onset of insulin resistance resulted in a shift in nutrient partitioning (change from glucose toward lipid consumption) and possibly futile cycling of those nutrients (energy expenditure due to a repeated elimination and composition of triglycerides). Furthermore, the reduction of thermogenic genes in BAT and reduced BAT glucose uptake were shown to impact metabolic health negatively. Still, additional experiments are needed to confirm this miR-mediated phenotype, for example, by doing glucose tolerance testing and assessment of whether glucose uptake is indeed due to insulin resistance or due to the observed reduced expression of *Glut4*. The fact that miR-224 is known to control FA metabolism of 3T3-L1 adipocytes (51), prevents 3T3-L1 apoptosis upon inflammation (52) and controls low-density lipoprotein (LDL) metabolism (via its target PCSK9) (53) is consistent with the notion that miRs coordinately regulate functionally related genes in similar processes (54). A striking example in which the miR-224/452 cluster simultaneously controls cellular metabolism was found in malignant melanomas, in which both miRs targeted thioredoxin interacting protein (TXNIP), a key transcription factor involved in redox regulation and tumor suppression (55). However, more studies with (synergistic) silencing of both miRs are needed to investigate their regulation of (adipocyte) metabolism in mice.

## Supplementary Material

Supplementary Materials

Supplementary Figure 1

Supplementary Figure 2

Supplementary Figure 3

Supplementary Figure 4

Supplementary Figure 5

Supplementary Table 1: Clinical characteristics of the female to male transgender cohort

Supplementary Table 2: Primer sequences for RT-qPCR

Supplementary Table 3

## Declaration of interest

The authors declare that there is no conflict of interest that could be perceived as prejudicing the impartiality of this study.

## Funding

This study was supported by funding provided by the Netherlands Heart Foundation http://dx.doi.org/10.13039/100002129 in the context of consortia: Queen of Hearts (A J V Z, B W F, 2013/T084), CVON-RECONNECT (A J V Z), and CVON-GENIUS-2 (P C N R), the European Fund for the Study of Diabetes and Boehringer Ingelheimhttp://dx.doi.org/10.13039/100001003 (to A J V Z and R B), the Dutch Kidney Foundation (KOLLF grant 16OKG16 to R B) and the Dutch Diabetes Research Foundation (ZonMw http://dx.doi.org/10.13039/501100001826, Doorbraak project 459001002 to A J V Z and B W F).

## Author contributions statement

B W F conducted experiments, acquired and analyzed data, and wrote the manuscript. M K, E N K, R W A L L, and S K acquired and analyzed data. J D, J L, G D T, A B, Y Y, and W S researched the data. R N, T J R, P C N R, A J V Z, and R B contributed to the discussion and reviewed and edited the manuscript. B W F, A J V Z, and R B are guarantors of this work and, as such, had full access to all the data in the study and responsibility for the integrity of the data and the accuracy of the data analyses. J M G J D and M K contributed equally. R B and A J v Z shared senior authorship.
