# Supplementary material for: Estradiol-driven metabolism in transwomen associates with reduced circulating extracellular vesicle microRNA-224/452
Source: Eur J Endocrinol. 2021 Aug 3;185(4):539–52. doi: 10.1530/EJE-21-0267 (PMC8436186; doi:10.1530/EJE-21-0267)
Supplement: Supplementary Materials [file supplementary_material.pdf]

Supplementary Methods

Patients

This study complied with the ethical principles of the Declaration of Helsinki and was conducted following approval by the Institutional Review Board of the Amsterdam University Medical center (Amsterdam UMC) and the Leiden University Medical Center (LUMC). All patients gave written informed consent. Transwomen received oral treatment with a daily dose of both 50 mg cyproteroneacetate (CPA) (Androcur®, Bayer) and 4 mg Estradiol Valerate (Progynova®, Bayer) or 100 µg/24h transdermal estradiol (Systen®, Janssen-Cilag) twice a week, as previously described (30). Paired plasma samples were obtained at the start of treatment and after one year of daily estrogen suppletion. Patient characteristics of the transwomen cohort 1 and 2 are displayed in Tables 1 and 2, respectively.

Plasma RNA isolation

Plasma RNA from each patient sample was isolated from 200 µL EDTA-plasma with 800 µL Trizol reagent (Invitrogen, The Netherlands) by using the RNeasy Micro Kit (Qiagen, The Netherlands) and an adapted protocol. In short, 160 µL chloroform was added to the Trizol sample and centrifuged for 15 min at 12,000 rpm. The aqueous phase volume was merged with 1.5x 100% ethanol, transferred to a MinElute Spin column (Qiagen) and centrifuged for 15s at 14,000 rpm. RNA was washed with 700 µL RWT buffer and centrifuged for 15s at 14,000 rpm. This was followed by two washing steps with 500 µL RPE buffer after which the column was centrifuged at 14,000 rpm. Total plasma RNA was eluted with 15 µL RNase-free water.

Library preparation and Next Generation Sequencing of Plasma miRs

Plasma miR sequencing was performed by Exiqon according to protocol. Briefly, plasma RNA was converted into microRNA NGS libraries using NEBNEXT library generation kit (New England Biolabs Inc.) according to the manufacturer's instructions. Each individual

RNA sample had adaptors ligated to its 3' and 5' ends and converted into cDNA. Then the cDNA was pre-amplified with specific primers containing sample specific indexes. After 18 cycle pre-PCR the libraries were purified on QiaQuick columns and the insert efficiency evaluated by Bioanalyzer 2100 instrument on high sensitivity DNA chip (Agilent Inc.). The miRNA cDNA libraries were size fractionated on a LabChip XT (Caliper Inc.) and a band representing adaptors and 15-40 bp insert excised using the manufacturer's instructions. Samples were then quantified using qPCR and concentration standards. Based on quality of the inserts and the concentration measurements the libraries were pooled in equimolar concentrations. The library pool(s) were finally quantified again with qPCR and optimal concentration of the library pool used to generate the clusters on the surface of a flow cell before sequencing using v3 sequencing methodology according to the manufacturer's instructions (Illumina Inc.). Samples were sequenced on the Illumina NextSeq 500 system. Experiments were conducted at Exiqon Services, Denmark. Sequencing data has been deposited in the Gene Expression Omnibus (GEO) and is accessible under GSE147966 (reviewer token for access: ahmfqeqglzwphcf).

Plasma EV isolation

Plasma EVs (70 nm) were isolated by applying 125 µL human plasma to a 3.64 mL Sepharose CL-2B size exclusion chromatography (SEC) column (31). Fractions (250 µL) were eluted with PBS and for each sample a newly packed column was used. Particle presence in different fractions was analysed using resistive pulse sensing (RPS, qNano, Izon Science, Christchurch, New Zealand) as described before (32) while protein presence in fractions 11-35 was confirmed using a BCA Protein Assay Kit (Pierce). EV fractions (fraction 1-10) were combined until a total volume of 2 mL eluate was collected and applied to an Amicon Ultra-4 Centrifugal Filter unit for concentration after which the presence of EVs was validated using nanoparticle tracking analysis (NTA, NS500; Nanosight, Amesbury, UK) and confirmed by Negative-Stain Transmission Electron Microscopy (TEM). EV fraction was

treated with proteinase-K (Thermo Fisher Scientific, The Netherlands) after which EV and protein fractions were combined with Trizol to isolate RNA.

qPCR validation of plasma miRNA, EV miRNAs and mRNA expression

Selected miRNAs were validated with quantitative PCR, using individual samples that comprised the pooled samples that were used for plasma miR sequencing. Taqman primers (Cat. 4427975, Thermo Fisher Scientific, Waltham, MA) were used according to manufacturer's protocols. Briefly, total plasma RNA and EV-RNA was reverse transcribed using TaqMan MicroRNA Reverse Transcription Kit (Cat. 4366596, Thermo Fisher Scientific). Obtained miRNA Ct-values were normalized to plasma levels of miR-16 and U6 small nuclear RNA and expression was quantified using the ΔΔCt method. RT-qPCR validation of NGS sequencing based differential gene expression was performed after 500 ng RNA was reverse transcribed using dinucleotide triphosphates (Invitrogen) and oligo(dT) (Invitrogen). Using a M-MLV First-Strand synthesis system (Invitrogen) cDNA was synthesized and validation of mRNA expression was carried out using SYBR Green Master Mix (Applied Biosystems). Target gene mRNA primer sequences are listed in Supplementary Table 2.

Animal experiments

Male C57Bl/6J mice (n=10 per group, age=8 weeks, Charles River Nederland, Maastricht, the Netherlands) were randomized in three groups and received two subcutaneous injections of 25 mg/kg locked nucleic acid (LNA) modified antisense miR-224 (antimiR-224), miR-452 (antimiR-452) or scrambled miR sequence (scramblemiR). Five days before injection, mice were individually housed in fully automated metabolic cages up and until the day of sacrifice and had access to *ad libitum* standard chow diet and drinking water *ad libitum*. Following antimiR injection blood was drawn to measure plasma insulin levels. Indirect calorimetry was performed in fully automated metabolic cages (LabMaster System, TSE Systems) during the 48 hours of the in vivo experiment. O_2 consumption (VO_2),

CO_2 production (VCO_2), and caloric intake were measured 48 hours and total EE was estimated from the VO_2 and resting energy requirement. Carbohydrate oxidation was calculated using the formula $((4.585*VCO_2) - (3.226*VO_2))*4$. Similarly, fat oxidation was calculated using the formula $((1.695*VO_2) - (1.701*VCO_2))*9$.

Plasma ELISA

Plasma insulin concentrations were measured by ELISA (Mercodia, 10-1247).

Clearance of radiolabeled glucose and lipoprotein-like particles

Glycerol tri[^{3}H]oleate-labeled lipoprotein-like triglyceride (TG)-rich emulsion particles (80 nm) were prepared as previously described (33) and [^{14}C]deoxyglucose ([^{14}C]DG) was added in a ^{3}H:^{14}C= 4:1 ratio. After 6 hours fasting, mice were injected with 200 μL of emulsion particles (1 mg TG per mouse) via the tail vein. After 15 minutes, organs were harvested and dissolved overnight at 56°C in Solvable (Perkin Elmer). Glycerol tri[^{3}H]oleate- and [^{14}C]DG-derived activity was quantified and expressed per gram of wet tissue weight.

Tissue histology and immunohistochemistry

Formalin-fixed interscapular BAT (iBAT), subcutaneous WAT (sWAT), and gonadal WAT (gWAT) were dehydrated in 70% EtOH, embedded in paraffin, and cut into 5-μm sections. Sections were stained with hematoxylin and eosin (H&E) using standard protocols. All sections were digitalized with Philips Digital Pathology Solutions (PHILIPS Electronics) for morphological measurement. White adipocyte size was quantified using ImageJ software (Version 1.50)

RNA-seq library preparation

The NEB Next Ultra Directional RNA Library Prep Kit for Illumina was used to process samples according to the manufacturer's protocol. Briefly, mRNA was isolated from total RNA using the oligo-dT magnetic beads. After fragmentation of the mRNA, a cDNA

synthesis was performed for ligation of with the sequencing adapters and PCR amplification of the resulting product.

Mapping and analysis of RNA-seq data

Mus musculus reference version GRCm38.p4 was used for alignment of samples. The reads were mapped to the reference sequences using a short-read aligner based on the Burrows-Wheeler Transform. The default mismatch rate of 2% (2 mismatches in a read of 100 bases) was used. Based on the mapped locations in the alignment file, the frequency of reads mapping to a transcript was determined. Read counts served as input for downstream RNA-seq differential expression analysis. The read counts were loaded into the DESeq package v 1.10.1, a statistical package within the R platform v2.15.3. Additionally, RPKM/FPKM (reads/fragments per kilobase of exon per million reads mapped) values were calculated. Sequencing data has been deposited in the Gene Expression Omnibus (GEO) and is accessible under GSE147966 (reviewer token for access: ahmfqeqglzwphcf).

Pathway Analysis

Pathway analyses was carried out using Ingenuity Pathway Analysis (IPA) software. Bias corrected p-values were determined, and pathways were selected with a p-value lower than 0.01.

Cell culture of murine brown adipocytes and 3T3-L1 white adipocytes

Brown preadipocytes were isolated from interscapular BAT depots of 5-week-old male C57BL/6J mice as previously described (34). Cells were reversibly immortalized using doxycycline-controlled expression of simian virus large T antigen and were grown confluent in growth medium consisting of DMEM/F-12 GlutaMAX (Thermo Fisher Scientific) supplemented with 10% heat-inactivated fetal call serum (Life Technologies Europe), 100

U/mL penicillin and 100 µg/mL streptomycin (Invitrogen). 100 ng/mL doxycycline (Sigma-Aldrich) was added to the growth medium for expansion of cells. Upon confluence, cells were differentiated in growth medium supplemented with 5.6 nM bovine insulin (Sigma-Aldrich), 126 µM sodium ascorbate (Sigma-Aldrich) and 1 µM of the peroxisome proliferator-activated receptor γ agonist rosiglitazone (Sigma-Aldrich), 10 mM HEPES (adjusted to pH 7.4 with NaOH). Experiments were performed on day 12-14 of differentiation. 3T3-L1 preadipocytes (Zenbio) were grown confluent in in growth medium consisting of DMEM, high glucose, 10% heat-inactivated FBS (Life Technologies Europe), penicillin and streptomycin. Two days after grown confluent, cells were differentiated in growth medium (DMEM / Ham's F-12 medium (1:1, v/v) supplemented with HEPES pH 7.4, 10% heat-inactivated FBS (Life Technologies Europe), human insulin, dexamethasone, penicillin and streptomycin, 3-isobutyl-1-methylxanthine (IBMX) and PPARγ agonist rosiglitazone.

Cell treatment

Differentiated 3T3-L1 adipocytes and differentiated brown adipocytes were incubated with 100 nM 17-ß estraodiol (E2758, Sigma Aldrich) for 48 hours. After 48 hours cells were washed PBS and combined with Trizol to isolate RNA.

Oxygen consumption and extracellular acidification of murine brown adipocytes

Oxygen consumption rate (OCR) and extracellular acidification rate (ECAR) were measured using the Seahorse XF96 analyzer (Agilent Technologies). LNA-antimiR-224 and -452 transfected murine brown adipocytes were cultured and differentiated in Seahorse cell culture plates following the protocol described above. One hour prior to the oxygen consumption measurements medium was replaced by Seahorse XF Base Medium (Agilent) with 5 mM glucose (Sigma-Aldrich), 0.5 mM sodium pyruvate, 1x GlutaMAX and 10% FBS (all from Life Technologies Europe). Basal respiration was measured three times, followed by three measurements after addition of 1.5 µM oligomycin (Cayman Chemical), 3 µM FCCP and 1 µM rotenone and antimycin A (all from Sigma-Aldrich). For extracellular acidification

measurements medium was replaced one hour prior to the run by Seahorse XF Base Medium (Agilent) with 1x GlutaMAX and 10% FBS (both from Life Technologies Europe) with pH adjusted to 7.4. Basal respiration was measured three times, followed by six measurements after addition of 10 mM glucose (Sigma-Aldrich), 1.5 µM oligomycin (Cayman Chemical) and 50 mM 2-deoxy-glucose (Sigma-Aldrich). OCR and ECAR values were adjusted for total protein using the BCA protein assay kit (Thermo Scientific Pierce) according to the manufacturer's instruction.

Mitotracker experiment

Immortalized murine brown adipocytes were transfected with LNA-antimiR-224 and -452 and incubated for 30 min with MitoTracker Green FM (125 nM; Thermo Fisher) and MitoTracker RedCMXRos (250 nM; Thermo Fisher) in DMEM/F12 (Sigma) without FBS. Hereafter, the medium was changed, and live cells were imaged using a confocal LSM (Leica TCS SP8, Leica Microsystems). Control cells were monitored to correct for potential photobleaching, and total cell fluorescence per condition was quantified using ImageJ.

Glucose uptake experiments

Glucose uptake by immortalized murine brown adipocytes transfected with LNA-antimiR-224 and -452 was performed using a glucose uptake colorimetric assay kit (Sigma-Aldrich/Merck, The Netherlands) according to the manufacturer's protocol. Briefly, transfected cells were starved in serum-free medium overnight, washed and then glucose starved. Subsequently, cells were stimulated with insulin after which glucose was added. Following incubation, cells were lyzed after which the lysate was combined with assay buffers and enzymes. Following the recycling amplification reaction absorbance was measured at 412 nm.

Statistics

Differential expression analysis of plasma microRNAs in the Next Generation Sequencing experiment was done using the EdgeR statistical software package (Bioconductor). For

normalization the trimmed mean of M-values method was used based on log-fold and absolute gene wise changes in expression levels between samples (TMM normalization). All other data are expressed as mean ± SEM. Variable distribution was tested using the Kolmogorov-Smirnov test for normal distribution. In addition, multivariable linear regression was used to adjust for possible confounders. Statistical analysis was performed with GraphPad software (La Jolla, CA) using two-tailed paired or unpaired Student's t-test or ANOVA with Bonferroni's post hoc test.

Study approval

These studies were approved by the Institutional Review Boards of both the VU University Medical Center (Amsterdam, The Netherlands) and the Leiden University Medical Center (Leiden, The Netherlands) and complied with the ethical principles of the Declaration of Helsinki. All patients gave written informed consent. All animal experiments and protocols were approved by the animal welfare committee of the veterinary authorities of the Leiden University Medical Center (Leiden, The Netherlands).
