# Supplementary material for: Estradiol-driven metabolism in transwomen associates with reduced circulating extracellular vesicle microRNA-224/452
Source: Eur J Endocrinol. 2021 Aug 3;185(4):539–52. doi: 10.1530/EJE-21-0267 (PMC8436186; doi:10.1530/EJE-21-0267)
Supplement: Supplementary Figure 1 [file supplementary_figure_1.pdf]

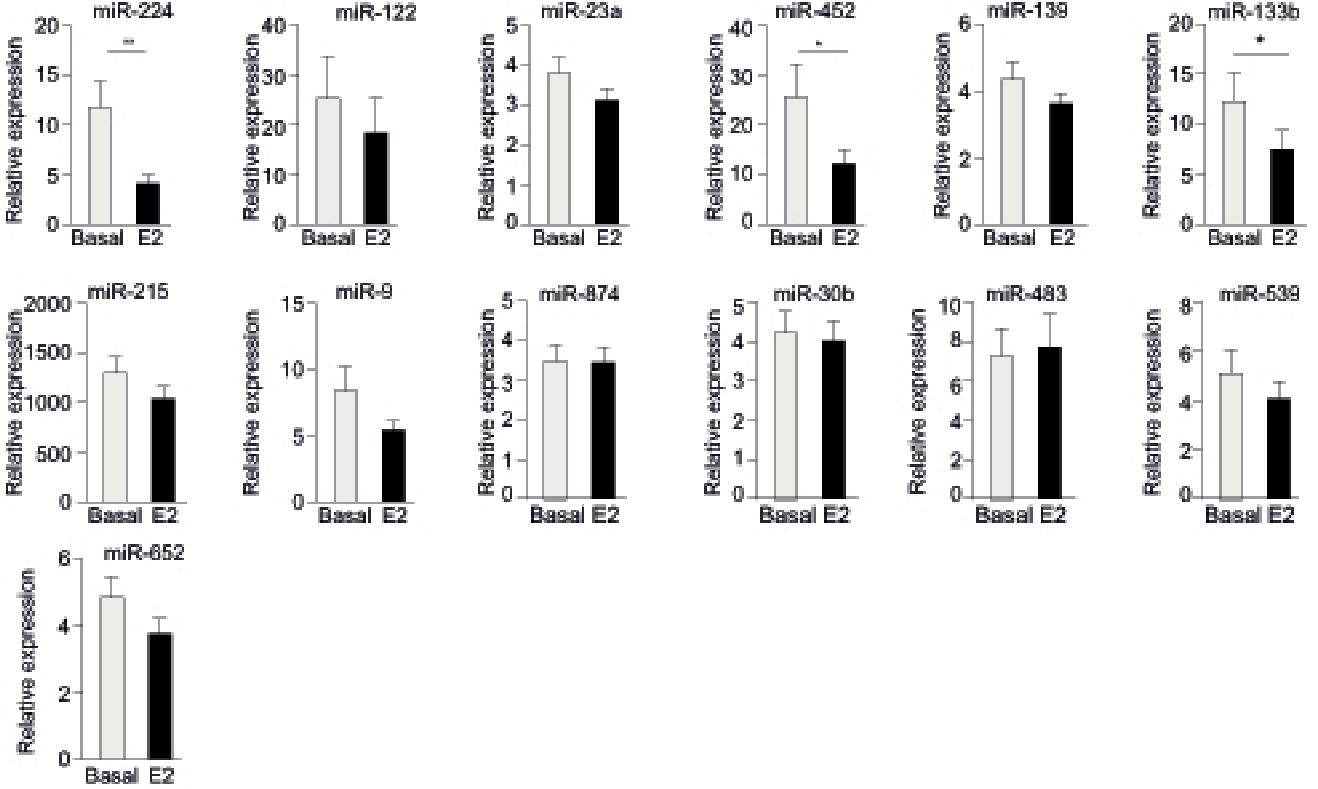

Supplementary Figure 1. RT-qPCR validation of 13 estrogen responsive circulating miRNAs in human plasma of male-to-female transgender persons (transwomen) N=20. Upon RT-qPCR validation of estrogen responsive miRNAs, miRNA-224, miRNA-452 and miRNA-133b display a significant decrease after one year estrogen treatment. E2, estrogen. *$p < 0.05$, **$p < 0.01$.
