# Supplementary material for: Estradiol-driven metabolism in transwomen associates with reduced circulating extracellular vesicle microRNA-224/452
Source: Eur J Endocrinol. 2021 Aug 3;185(4):539–52. doi: 10.1530/EJE-21-0267 (PMC8436186; doi:10.1530/EJE-21-0267)
Supplement: Supplementary Figure 2 [file supplementary_figure_2.pdf]

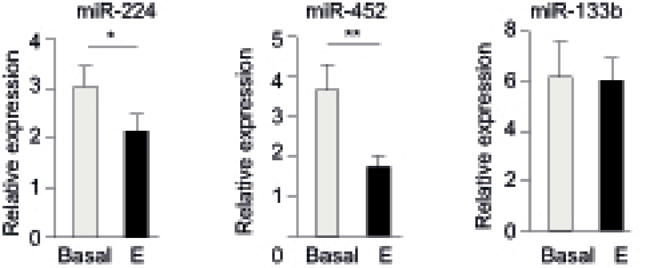

Supplementary Figure 2. RT-PCR validation of plasma miR-224, miR-452 and mir-133b in transwomen (n=30). E: estradiol,*p< 0.05, **p< 0.01 according to a paired student T-test.
