# Supplementary material for: Estradiol-driven metabolism in transwomen associates with reduced circulating extracellular vesicle microRNA-224/452
Source: Eur J Endocrinol. 2021 Aug 3;185(4):539–52. doi: 10.1530/EJE-21-0267 (PMC8436186; doi:10.1530/EJE-21-0267)
Supplement: Supplementary Figure 3 [file supplementary_figure_3.pdf]

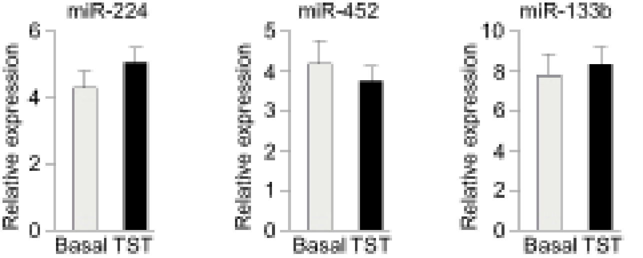

Supplementary Figure 3. RT-PCR validation of miR-224, miR-452 and miR-133b in female-to-male transgenders (transmen) n=50. TST, testosterone. *$p < 0.05$, **$p < 0.01$ according to a paired student T-test.
