# Supplementary material for: Estradiol-driven metabolism in transwomen associates with reduced circulating extracellular vesicle microRNA-224/452
Source: Eur J Endocrinol. 2021 Aug 3;185(4):539–52. doi: 10.1530/EJE-21-0267 (PMC8436186; doi:10.1530/EJE-21-0267)
Supplement: Supplementary Figure 4 [file supplementary_figure_4.pdf]

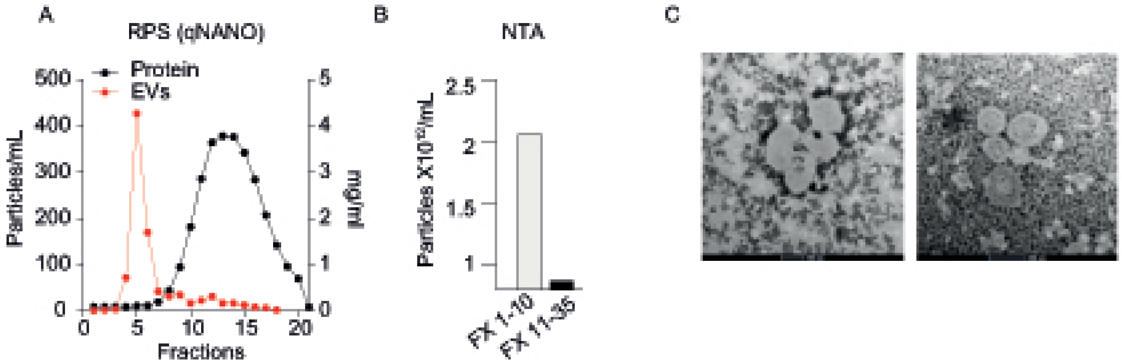

Supplementary Figure 4. (A) EV concentration in different fractions was analysed using resistive pulse sensing (RPS) while protein presence in each fraction was assessed with a BCA protein assay. (B) After EV fractions were combined to a total volume of 2 mL, EV presence was validated using nanoparticle tracking analysis (NTA). (C) EV presence was validated with transmission electron microscopy
