# Supplementary material for: Estradiol-driven metabolism in transwomen associates with reduced circulating extracellular vesicle microRNA-224/452
Source: Eur J Endocrinol. 2021 Aug 3;185(4):539–52. doi: 10.1530/EJE-21-0267 (PMC8436186; doi:10.1530/EJE-21-0267)
Supplement: Supplementary Figure 5 [file supplementary_figure_5.pdf]

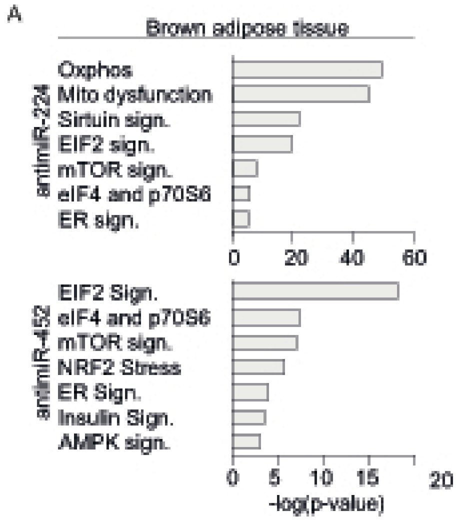

Supplementary Figure 5. Ingenuity pathway analysis (IPA) derived top canonical pathways next-generation sequencing (NGS)-based differential gene expression (FPKM, p<0.05, n= 4). (A) In BAT mitochondrial dysfunction and insulin receptor signalling were significant top canonical pathways in antimiR-224 and antimiR-452 treated BAT respectively. (B) In antimiR-224 treated WAT tissue, AMPK signalling (regulates adipocyte metabolism) and STAT3 (controls lipogenesis and adipocyte hypertrophy) were affected, while antimiR-452 treatment affected the HIPPO-pathway (adipocyte differentiation) and insulin receptor signalling.
