# Supplementary material for: Estradiol-driven metabolism in transwomen associates with reduced circulating extracellular vesicle microRNA-224/452
Source: Eur J Endocrinol. 2021 Aug 3;185(4):539–52. doi: 10.1530/EJE-21-0267 (PMC8436186; doi:10.1530/EJE-21-0267)
Supplement: Supplementary Table 1: Clinical characteristics of the female to male transgender cohort [file supplementary_table_1.pdf]

Supplementary Table 1: Clinical characteristics of the female to male transgender cohort

| | Baseline | Testosterone | p-value |
|---|---|---|---|
| Age (years) | 26.9 ± 13.3 | 26.9 | |
| BMI (kg/m2) | 25 ± 4.9 | 23.8 ± 7.2 | 0.623 |
| Oestrogen (pmol/L) | 259.4 ± 329 | 254.1 ± 249.3 | 0.985 |
| Testosterone (nmol/L) | 1.32 ± 0.6 | 38.4 ± 29.1 | 0.001 |
| Systolic blood pressure (mm Hg) | 125 ± 10 | 124 ± 12 | 0.696 |
| Diastolic blood pressure (mm Hg) | 76 ± 11 | 75 ± 9 | 0.421 |
| Hemoglobin (mmol/L) | 8.5 ± 0.7 | 9.6 ± 0.6 | 0.001 |
| Hematocrit (L/L) | 0.41 ± 0.03 | 0.47 ± 0.03 | 0.001 |
| Glucose (mmol/L) | 5.2 ± 0.56 | 5.1 ± 1.40 | 0.373 |
| Insulin (pmol/L) | 70.7 ± 39 | 61.4 ± 45.4 | 0.834 |
| Creatinine (umol/L) | 67 ± 9.3 | 81.9 ± 11.1 | 0.001 |
| Cholesterol (mmol/L) | 4.3 ± 1.11 | 4.3 ± 0.83 | 0.864 |
| Triglyceriden (mmol/L) | 0.93 ± 0.57 | 1.13 ± 0.93 | 0.029 |
| HDL (mmol/L) | 1.51 ± 0.39 | 1.26 ± 0.30 | 0.001 |
