# Supplementary material for: Estradiol-driven metabolism in transwomen associates with reduced circulating extracellular vesicle microRNA-224/452
Source: Eur J Endocrinol. 2021 Aug 3;185(4):539–52. doi: 10.1530/EJE-21-0267 (PMC8436186; doi:10.1530/EJE-21-0267)
Supplement: Supplementary Table 2: Primer sequences for RT-qPCR [file supplementary_table_2.pdf]

Supplementary Table 2: Primer sequences for RT-qPCR

Gene	Primer sequence	
Acc1	Forward<br>Reverse	5'- TGTCCACCCAAGCATTTCTTC -3'<br>5'- ATCCAACACCAGTTCAGTATACGT -3'
Acsl1	Forward<br>Reverse	5'-TGCCAGAGCTGATTGACATTC-3'<br>5'-GGCATACCAGAAGGTGGTGAG-3'
Adam10	Forward<br>Reverse	5'- GTGCCAAACGAGCAGTCTCA-3'<br>5'- ATTCGTAGGTTGAACTGTCTTCC-3'
Apoc1	Forward<br>Reverse	5'-GGGCAGCCATTGAACATATC-3'<br>5'-ACCTGGCACATTACGTGGAT-3'
Arf6	Forward<br>Reverse	5'- ATCTTCGGGAACAAGGAAATGC-3'<br>5'- GGATGGTGGTCACCGATTGG-3'
Chop	Forward<br>Reverse	5'-ACCTTCACTACTCTTGACCCTG-3'<br>5'-GATGTGCGTGTGACCTCTGT-3'
Cidea	Forward<br>Reverse	5'-CTCGGCTGTCTCAATGTCAA-3'<br>5'-CCGCATAGACCAGGAACTGT-3'
Cntf	Forward<br>Reverse	5'- AGCAAGGAAGATTCGTTCAGAC-3'<br>5'- TCAGTCATCTCACTCCAGCGA-3'
Cox16b1	Forward<br>Reverse	5'- GGTGATGTCTCCGTGTGTGAG<br>5'- TGCCTTCAGCTATGCGGTC
Cox17	Forward<br>Reverse	5'- TGCCCGGAAACCAAGAAGG<br>5'- AAATCCCAGTGCCCTCATGC
Cpt1α	Forward<br>Reverse	5'-GAGACTTCCAACGCATGACA-3'<br>5'-ATGGGTTGGGGTGATGTAGA-3'
Cxcl14	Forward<br>Reverse	5'- GAAGATGGTTATCGTCACCACC-3'<br>5'- CGTTCCAGGCATTGTACCACT-3'
Eif3e	Forward<br>Reverse	5'- ATGTGGTAATGGGCAACAATGC-3'<br>5'- TGGCCAACATTTGGCTTCTA-3'
Glut1	Forward<br>Reverse	5'-GACGGGCCGCCTCATGTTGG -3'<br>5'-GCTCTCCGTAGCGGTGGTTCC -3'
Glut4	Forward<br>Reverse	5'-CTATTCAACCAGCATCTTCGAG -3'<br>5'-CTACTAAGAGCACCGAGACC -3'
Gpr120	Forward<br>Reverse	5'- CCCCTCTGCATCTTGTTCC -3'<br>5'- GATTTCTCCTATGCGGTTGG -3'
Hsl	Forward<br>Reverse	5'- CGAGACAGGCCTCAGTGTGA -3'<br>5'- TCTGGGTCTATGGCGAATCG -3'
Id3	Forward<br>Reverse	5'- CTGTCGGAACGTAGCCTGG<br>5'- GTGGTTCATGTCGTCCAAGAG
Irs1	Forward<br>Reverse	5'-CGATGGCTTCTCAGACGTG-3'<br>5'-CAGCCCGCTTGTTGATGTTG-3'
Irs2	Forward<br>Reverse	5'- AACCTGAAACCTAAGGGACTGG-3'<br>5'- CGGCGAATGTTCATAAGCTGC-3'
Mex3c	Forward<br>Reverse	5'- TGTCACTGGACGGAAAGAAGA-3'<br>5'- TGCACGAATCATAGAAAAGTGCT-3'
Mlxipl	Forward<br>Reverse	5'- CCCTCAGACACCCACATCTT -3'<br>5'- TCAGAAAGGGGTTGGGATCC -3'
Ndufa1	Forward<br>Reverse	5'-GCCTTACCACCTGTGTCAGTG<br>5'- CCCATAGCTGTGAGTGCGAG
Nr4a1	Forward<br>Reverse	5'- GAGTTCGGCAAGCCTACCAT<br>5'- GTGTACCCGTCCATGAAGGTG
Pgc1α	Forward<br>Reverse	5'- GATGGCACGCAGCCCTAT -3'<br>5'- CTCGACACGGAGAGTTAAAGGAA -3'
Pnrc2	Forward<br>Reverse	5'- GGTGGCGGAGAGAGGTATAAC-3'<br>5'- ATGAGCAATCTTCGTCTGGGA-3'

Sdc4	Forward<br>Reverse	5’- GCTGGTGTACCGCATGAAGAA-3’<br>5’- ATGCGTAGAACTCATTGGTGG-3’
Sema3c	Forward<br>Reverse	5’- ATGGCATTCCGGGCGATTT-3’<br>5’- GGTTTTGGTTTCTCGAAGCTCA-3’
Sod1	Forward<br>Reverse	5’- AACCAGTTGTGTTGTCAGGAC<br>5’- CCACCATGTTTCTTAGAGTGAGG
Srebp1c	Forward<br>Reverse	5’- ATGCCATGGGCAAGTACACA -3’<br>5’- ATAGCATCTCCTGCGCACTC -3’
Stard4	Forward<br>Reverse	5’- GATGAGTGGCGAGTTGCCAA-3’<br>5’- TGGGCGGATATGGTCTATTACA-3’
Tgif1	Forward<br>Reverse	5’- CTGTCAGCCGATTGGTGTG-3’<br>5’- TGGGTACACGAGAGAGGTGTC-3’
Ucp2	Forward<br>Reverse	5’-CACTTTCCCTCTGGATACCG-3’<br>5’-GCTCTGAGCCCTTGGTGTAG-3’
Ucp3	Forward<br>Reverse	5’-CCTACGACATCATCAAGGAGAA-3’<br>5’-GCCACCATCTTCAGCATACA-3’
